# Evaluating an app-guided self-test for influenza: lessons learned for improving the feasibility of study designs to evaluate self-tests for respiratory viruses

**DOI:** 10.1186/s12879-021-06314-1

**Published:** 2021-06-29

**Authors:** Monica L. Zigman Suchsland, Ivan Rahmatullah, Barry Lutz, Victoria Lyon, Shichu Huang, Enos Kline, Chelsey Graham, Shawna Cooper, Philip Su, Sam Smedinghoff, Helen Y. Chu, Kara Sewalk, John S. Brownstein, Matthew J. Thompson, on behalf of Seattle Flu Study investigators

**Affiliations:** 1 University of Washington, 4225 Roosevelt Way NE Ste 308, Seattle, WA 98105-6099 USA; 2 Massachusetts Institute of Technology, Cambridge, MA USA; 3 Audere, Seattle, WA USA; 4 Boston Children’s Hospital, Boston, MA USA

**Keywords:** Influenza, Self-test, Mobile application, Accuracy, Rapid diagnostic test

## Abstract

**Background:**

Seasonal influenza leads to significant morbidity and mortality. Rapid self-tests could improve access to influenza testing in community settings. We aimed to evaluate the diagnostic accuracy of a mobile app-guided influenza rapid self-test for adults with influenza like illness (ILI), and identify optimal methods for conducting accuracy studies for home-based assays for influenza and other respiratory viruses.

**Methods:**

This cross-sectional study recruited adults who self-reported ILI online. Participants downloaded a mobile app, which guided them through two low nasal swab self-samples. Participants tested the index swab using a lateral flow assay. Test accuracy results were compared to the reference swab tested in a research laboratory for influenza A/B using a molecular assay.

**Results:**

Analysis included 739 participants, 80% were 25–64 years of age, 79% female, and 73% white. Influenza positivity was 5.9% based on the laboratory reference test. Of those who started their test, 92% reported a self-test result. The sensitivity and specificity of participants’ interpretation of the test result compared to the laboratory reference standard were 14% (95%CI 5–28%) and 90% (95%CI 87–92%), respectively.

**Conclusions:**

A mobile app facilitated study procedures to determine the accuracy of a home based test for influenza, however, test sensitivity was low. Recruiting individuals outside clinical settings who self-report ILI symptoms may lead to lower rates of influenza and/or less severe disease. Earlier identification of study subjects within 48 h of symptom onset through inclusion criteria and rapid shipping of tests or pre-positioning tests is needed to allow self-testing earlier in the course of illness, when viral load is higher.

**Supplementary Information:**

The online version contains supplementary material available at 10.1186/s12879-021-06314-1.

## Background

Seasonal influenza poses a substantial societal burden through morbidity and mortality annually. In the United States (US) alone, there are 37.4–42.9 million infections per year, with approximately half leading to health care visits and 36,400–61,200 deaths [1]. Health impact aside, the overall economic burden of seasonal influenza in the US from medical costs and indirect costs such as lost productivity is estimated to be $11.2 billion annually [2]. One main issue in effectively managing illness is differentiating influenza from other viral or bacterial respiratory tract infections [3]. This uncertainty can lead to over-prescribing of antibiotics (for presumed bacterial infections), as well as under-treatment of influenza since anti-influenza treatment is generally only recommended within 48 h of symptom onset except in severe cases [4, 5]. This diagnostic challenge has major implications for influenza management because many people do not seek medical care for influenza like illnesses (ILI) until at least 2 days after symptom onset, thus missing the treatment window and allowing greater time for viral spread due to delayed initiation of infection control measures [5, 6].

In the US, current clinical guidelines in ambulatory settings recommend testing for influenza in patients with ILI symptoms who are at risk for complications, or if testing will influence management such as starting antiviral treatment [7]. Typically testing involves an upper respiratory tract specimen (usually mid-turbinate or nasopharyngeal swab) by a health care professional, which is used to detect influenza either using on-site point of care or central laboratory assays. However, all current influenza tests in the US require an individual to visit a health care facility or pharmacy in order for a sample to be obtained. This requirement may act as a barrier to testing for some individuals, such as those who experience difficulties accessing healthcare due to out of pocket expenses, or constraints from work, school or childcare responsibilities.

The impact of seasonal influenza has prompted efforts to develop rapid diagnostic tests (RDTs) that individuals could conduct at home, unsupervised by healthcare professionals. However, demonstrating the accuracy of self-test RDTs for home use involves several challenges, including ensuring that an adequate nasal specimen is obtained, correctly following the instructions to perform the RDT, interpreting the test results, and understanding the implications or actions needed based on the test result. While there is robust evidence that viral detection from self-swabbing of the nose is equivalent to that of health care professionals (and is the current recommendation from the FDA for SARS-CoV-2 specimen collection) [8–11], often guided by web-based or mobile tools [12, 13], there is very limited evidence currently to support the entire self-testing process for influenza.

We developed a study to determine the accuracy of a mobile app guided self-test using a lateral flow RDT for influenza, compared to a reference test of a self-swab sample sent to a research laboratory. The lessons learned from the methods employed have implications for conduct of similar comparative accuracy studies of self-tests for influenza and other respiratory pathogens.

## Methods

### Study design

We conducted an observational study to investigate the accuracy of an app-guided at-home test for influenza among adults experiencing influenza-like illness (ILI). This study was approved by the University of Washington Human Subjects Division (STUDY00006388).

### Study population

A convenience sample of participants were recruited from March 4, 2019 – April 26, 2019. Eligible participants were 18 years and older, had an iPhone/iPad, and had ILI defined as presence of a cough and at least one or more of the following symptoms: fever, chills or sweats, muscle/body aches, or feeling tired/more tired than usual (Additional file 2) [14, 15]. Assuming a flu positivity rate of 10% and an attrition rate of return of the reference test of 50%, the estimated sample size needed to recruit 150 flu positive participants was 3000.

### Recruitment

Participants were recruited within the continental US through emails to members of the Flu Near You influenza surveillance platform (flunearyou.org, Boston, MA), and through targeted advertisements on websites and social media platforms. Alaska and Hawaii were excluded due to potentially longer shipping times. Advertisements and recruitment emails encouraged individuals to download a study-specific app to determine eligibility for participation.

### Mobile application

An iOS application (flu@home) was developed (Audere, Seattle, WA, Additional file 1) that served multiple functions: eligibility screening, obtaining electronic consent, administering the study questionnaire, step-by-step instructions for obtaining a low nasal swab and conducting a lateral flow test, and details on return of a second nasal swab to the research team for in-lab testing. The flu@home app also contained general information about influenza and directed participants to appropriate publicly-available patient resources about influenza.

### Self-testing methods and quantitative data collection

After downloading the flu@home app onto their device, the app assessed eligibility, based on study inclusion criteria, through two specific questions (Additional file 2). Interested participants could take the eligibility survey once every 24 h to ascertain eligibility. Eligible participants were then guided through informed consent which was recorded in the app and a copy of the consent form was emailed to the participant if requested. Next, the app arranged for free next-day delivery (for orders before 1 pm and 2 day delivery for orders placed after 1 pm) of a self-test kit to the user’s home address. Orders placed over the weekend were not processed until Monday and the shipping service did not deliver to most residential addresses on Saturday. Once the test kit arrived, the app provided directions to complete the QuickVue Influenza A + B RDT (Quidel Corporation, San Diego, CA). This involved collecting a low nasal swab from both nostrils using a 3 in. foam tipped swab (Quidel Corporation, San Diego, CA), inserting the swab into a small tube containing reagent solution to disrupt the virus, stirring 4 times, and leaving it in the solution for 1 min. The user was then instructed to remove the swab by squeezing it against the side of the tube, thereby extracting the liquid from the swab into the tube. The lateral flow test strip was inserted into the tube, with instructions to leave it in the solution for 10 min which was timed by the app. During this test processing time, the app prompted participants to answer a series of questions on 9 symptoms (including their date of onset and severity) to assess how they felt when they took the test, as well as smoking history, contact with individuals with respiratory illness, underlying medical conditions, history of influenza vaccination, and impact of illness on daily activities (Additional file 2). The questionnaire was developed specifically for this study. The app notified the participant when 10 min had elapsed, and asked them to remove the test strip from the tube and visually examine the test strip for the presence and position of lines. They were first asked whether they saw a blue line in the middle of the test strip. If they indicated there was no blue line, they were not asked further questions about what they saw on the test strip, as a test strip without a control line (blue line) was considered invalid. All participants indicating they did see a blue control line were asked whether there were any red lines (test lines) showing on their test strip and presented with the following 4 options in the app: a) No red lines, b) Yes, above the blue line, c) Yes, below the blue line, or d) Yes, above and below the blue line. While these 4 options indicated a) a valid negative result, b) a valid influenza A result, c) a valid influenza B result and, d) a valid influenza A and B result, respectively, these interpretations were not provided to study participants or to those at the central laboratory conducting the reference test. Participants took a picture of the test strip using the app, which was available for the research team to later review, and participants were asked to rate how well they thought they performed the rapid test and reference sample collection.

### Reference testing

Participants were then instructed to collect a second nasal swab using an identical technique to the first swab, and place it in universal transport medium (UTM) (Becton Dickinson, Franklin Lakes, NJ); the app provided instructions on packaging and shipping of the sample via prepaid priority mail to the research laboratory. The packaging of returned samples was inspected on receipt at the lab. In accordance with expert guidelines for influenza testing, a polymerase chain reaction (PCR) diagnostic (X-pert Xpress Flu/RSV Assay, Cepheid Inc., Sunnyvale, CA) was selected as the reference standard. The reference swab was tested for influenza A and B by PCR [7]. The Cepheid test is an FDA-cleared CLIA-waived assay and instrument that extracts and detects RNA from influenza A (two targets), influenza B, and RSV, as well as a sample process control. Test results and cycle threshold (Ct) values for each target were calculated and interpreted by instrument embedded algorithms and exported for use in this study. Results of the reference test were not shared with the participant. Test strip images captured by participants were interpreted by two members of the research team blinded to each other’s interpretation and blinded to the reference test results. Discrepancies in test image interpretation were reviewed with two other research members who were also blinded to any other interpretations, and to the reference test results.

### Compensation

Study participants were given a gift card on completion of the study, defined as receipt of the reference sample at the research laboratory. The initial compensation for participation was $50. The study advertisement was posted to external coupon/cash back websites creating a sudden increase in participant responses that exceeded research team ability to supply test kits. In response, these websites were blocked and we reduced the compensation amount to $25 on March 17, 2019.

### Data analysis

We calculated the numbers of study participants who completed each step in the app (download, eligibility, consent, ordered kit, scanned kit bar code, questionnaire completion, RDT image capture, and app completion), as well as receipt of reference samples in the research lab, and successful analysis of the reference test. Time to completion of study steps, missing values, and error rates of packaged samples were tracked and reported. Summary statistics of demographics, risk factors, potential exposures, symptoms, and symptom severity were calculated. Comparisons between PCR+ and PCR- samples were made using Chi-square (or where appropriate Fisher’s exact) tests. Comparisons were statistically significant at *P* ≤ 0.05.

We calculated sensitivity, specificity, positive and negative likelihood ratios (and 95% Confidence Intervals (95%CI)) of the test strip results interpreted by participants compared to the presence of influenza detected by the reference test. We also calculated the accuracy of research staff interpretation of the image of the test strip, compared to the presence of influenza in the reference test. Subgroup analyses explored accuracy of the test strip compared to participants who completed reference testing within 4 days of ordering the test kit, tests that were conducted by participants within 3 days of reported symptom onset, and in reference samples that were received without discolored UTM fluid. Analyses were conducted in RStudio (Version: Desktop 1.2.5033, RStudio, Inc., Boston, MA). The study is reported in accordance with STARD guidelines.

## Results

### Participant recruitment and study completion rates

**Fig. 1:**
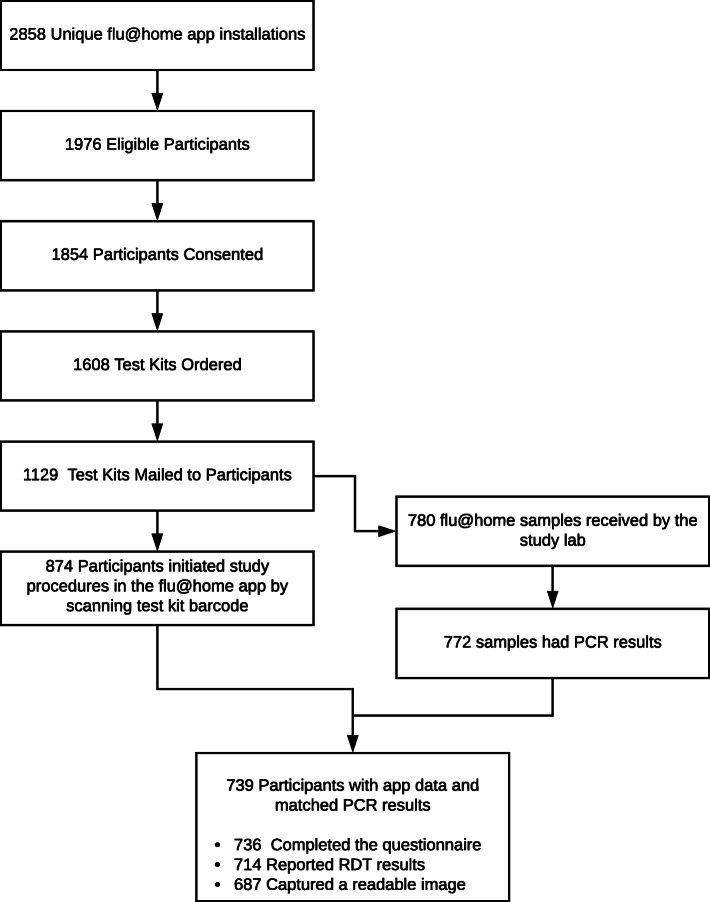
Flow of study participants

There were a total of 2858 unique installations of the flu@home app (Fig. 1). Each app/device allowed multiple participants to complete study procedures (i.e. people in the same household could use the same app), of whom 1976 participants met eligibility criteria, 1853 provided consent, and 1608 ordered a test kit. Of these, the research team mailed 1129 test kits; 450 test kits were ordered but could not be fulfilled due to limited test kit inventory at the time of ordering. Other reasons kits were not shipped included addresses in Alaska or Hawaii, or undeliverable addresses.

There were 874 participants who initiated study procedures in the app by scanning a barcode located on the test kit, 811 reported an RDT result in the app, and 780 samples were shipped back to the lab. Eight of 780 that were received at the lab could not be tested and therefore do not have PCR results. Of the remaining 772 samples, we were able to match a total of 739 out of 772 PCR results from reference samples to flu@home app records. PCR results for 33 records could not be matched to flu@home app records because: samples (19) returned to the lab did not have identification barcodes, or samples (14) returned to the lab did not have a barcode that matched the barcode in the app due to incorrect manual entry or scanning the wrong barcode (such as the shipping label) by the participant. The remainder of the results presented here refer to the final study sample of 739 matched records.

Participants were recruited from 39 states, and of responses available (*n* = 552), the majority were recruited through online advertisements (218, 29.5%), online searches (118, 15.9%), referred through friends (91, 12.3%), with the remainder recruited from Flu Near You, the App store or other sources (Additional file 3).

### Description of participants, risk factors for influenza, and symptoms

The majority of participants were between 25 and 64 years of age (80.6%), female (79%), and white (73%) (Table 1). Most were non-smokers (81.2%) and had either private (57.8%) or government insurance (34.1%). A minority (16.4%) were currently taking antibiotics or antiviral medications, and most (59.7%) had been in contact with a person who appeared to the participant to have a cold in the past week. Influenza was detected in the reference test on 43 (5.9%) participants (41 influenza A, 2 influenza B). Influenza positivity on PCR, was significantly associated with current illness interfering with daily activity (90.7% vs 67.2%, *p* = 0.002).

**Table 1:** Characteristics of study participants and association with influenza status

Characteristics N (%)	Overall (***N*** = 739)	PCR+ (***N*** = 43)	PCR- (***N*** = 696)	***p***-value
***Demographics***				
**Age**				
18 to 24	86 (11.6)	0 (0)	86 (12.4)	**0.002**
25 to 34	202 (27.3)	12 (27.9)	190 (27.3)	
35 to 44	191 (25.8)	19 (44.2)	172 (24.7)	
45 to 64	203 (27.5)	12 (27.9)	191 (27.4)	
65 and older	57 (7.7)	0 (0)	57 (8.2)	
**Sex (*n*** **= 736)**				
Female	584 (79.0)	35 (81.4)	549 (78.9)	0.865
Male	150 (20.3)	8 (18.6)	142 (20.4)	
Other	2 (0.3)	0 (0)	2 (0.3)	
**Race (*n*** **= 730)**				
White	539 (73.0)	35 (81.4)	504 (72.4)	0.289
Black	59 (8.0)	1 (2.3)	58 (8.3)	
Asian	62 (8.4)	2 (4.7)	60 (8.6)	
Native (American Indian, Alaska Native, native Hawaiian)	6 (0.8)	0 (0)	6 (0.9)	
Other	30 (4.1)	4 (9.3)	26 (3.7)	
Mixed race	34 (4.6)	1 (2.3)	33 (4.7)	
**Ethnicity (Hispanic or Latino) (*n*** **= 733)**				
No	654 (88.5)	38 (88.4)	616 (88.5)	1.0
Yes	79 (10.7)	4 (9.3)	75 (10.8)	
***Exposure or Risk Factor***				
**Health Insurance (*n*** **= 737)**				
Government	252 (34.1)	14 (32.6)	238 (34.2)	0.963
No Insurance	58 (7.8)	3 (6.9)	55 (7.9)	
Private	427 (57.8)	26 (60.5)	401 (57.6)	
**Received flu shot in the last year (*n*** **= 715)**				
No	359 (48.6)	21 (48.8)	338 (48.6)	0.977
Yes	356 (48.2)	22 (51.2)	334 (47.9)	
**Currently smokes tobacco (*n*** **= 734)**				
No	600 (81.2)	39 (90.7)	561 (80.6)	0.172
Yes	134 (18.1)	4 (9.3)	130 (18.7)	
**Currently taking antibiotics or antivirals (*n*** **= 729)**				
No	608 (82.3)	32 (74.4)	576 (82.5)	0.149
Yes	121 (16.4)	11 (25.6)	110 (15.8)	
**Contact with a person who seemed to have a cold in the past week (*n*** **= 734)**				
Don’t know	184 (24.9)	9 (20.9)	175 (25.1)	
No	109 (14.7)	8 (18.6)	101 (14.5)	0.735
Yes	441 (59.7)	26 (60.5)	415 (59.6)	
**If yes to contact with a sick person did they have a cough or sneeze (*n*** **= 441)**				
Don’t know	15 (3.4)	1 (3.4)	14 (3.4)	
No	14 (3.2)	3 (11.5)	11 (2.6)	**0.039**
Yes	406 (92.0)	21 (80.8)	385 (92.8)	
**Current illness interferes with daily activity (*n*** **= 737)**				
No	230 (31.1)	4 (9.3)	226 (32.5)	**0.002**
Yes	507 (68.6)	39 (90.7)	468 (67.2)	

The majority of individuals (78.6%) reported 6 or more symptoms (Additional file 4). A total of 70% of PCR+ participants reported 8 or 9 symptoms and 11.6% reported 5 or fewer symptoms, compared to 39.1 and 21.7% of PCR- participants respectively (*p* = 0.041). The most frequently reported symptoms overall were fatigue (92.5%), cough (90.8%) or runny nose (88.9%) (Table 2). Cough was a required symptom for study eligibility but 9.2% of participants did not report cough in the symptom questionnaire collected after enrollment. Fever and chills/sweats were significantly associated with PCR positivity (*p* = 0.0004 and *p* = 0.0003, respectively). Reported severity of chills/sweats, fatigue, and fever was greater in PCR+ than PCR- participants. Participants with influenza were also more likely to report moderate or severe fever, cough, fatigue, muscle or body aches, headache or shortness of breath, and less likely to report severe sore throat or runny nose than those without influenza, but none of these associations were statistically significant (Additional file 5).

**Table 2:** Reported influenza symptoms, reported symptom severity, and association with influenza status

Symptoms N (%)	Overall (***N*** = 739)	PCR + (***N*** = 43)	PCR – (***N*** = 696)	***p***-value
Fatigue	686 (92.5)	42 (97.7)	644 (92.5)	0.334
Cough	671 (90.8)	42 (97.7)	629 (90.4)	0.163
Runny Nose	657 (88.9)	39 (90.7)	618 (88.8)	0.892
Headache	615 (83.2)	35 (81.4)	580 (83.4)	0.904
Muscle or Body Aches	581 (78.6)	37 (86.1)	544 (78.2)	0.301
Sore throat	570 (77.1)	35 (81.0)	535 (76.9)	0.617
Chills or Sweat	518 (70.1)	41 (95.4)	477 (68.5)	**0.0003**
Fever	440 (59.3)	37 (86.1)	403 (57.9)	**0.0004**
Shortness of Breath	333 (45.1)	25 (58.1)	308 (44.2)	0.105

### Timing of illness and completion of study procedures

The number of days that elapsed between ordering the test kit and starting the home test (indicated by scanning the test kit barcode in the app), ranged from zero (same day) to 40 days; 617 (83.4%) participants started study procedures within 4 days, and 474 (64.1%) within 2 days of ordering the test kit, (Table 3). The time from starting the home test to receipt of the reference swab sample at the research lab varied from zero to 28 days; 449 (60.9%) were received within 4 days, and 289 (39.1%) within 5–28 days (Table 3). Only 4.7% participants completed the RDT within 2 days of symptom onset, 15.7% completed it within 3 days, and 587 (79.4%) completed the RDT 4 or more days after the start of their earliest-reported symptom. A higher percent of participants with influenza had symptom onset within 3 days of taking the test compared to those without influenza (*p* = 0.024) (Additional file 6).

**Table 3:** Time taken to complete required steps in the study

N (%)	Overall (***N*** = 739)	PCR+ (***N*** = 43)	PCR- (***N*** = 696)	***p***-value
**Number of days between ordering a test kit, shipping/delivery, and beginning testing procedures at home**				
No data	28 (3.8)	2 (4.7)	26 (3.7)	0.191
0–1 day	171 (23.1)	10 (23.3)	161 (23.2)	
2 days	303 (41.0)	23 (53.4)	280 (40.3)	
3 days	88 (11.9)	4 (9.3)	84 (11.9)	
4 days	55 (7.4)	3 (6.9)	52 (7.5)	
5–40 days	94 (12.7)	1 (2.3)	93 (13.4)	
**Number of days from starting test procedure at home to receipt of reference sample at the study lab**^**a**^				
No data	1 (0.1)	0	1 (0.1)	0.2183
0–1 days	9 (1.2)	1 (2.3)	8 (1.1)	
2 days	96 (12.9)	8 (18.6)	88 (12.7)	
3 days	176 (23.8)	7 (16.3)	169 (24.2)	
4 days	168 (22.7)	14 (32.6)	154 (22.2)	
5–28 days	289 (39.1)	13 (30.2)	276 (39.7)	

^a^Includes package pick-up/dropoff and shipping time to study lab

### Self-reported errors in test performance and errors in reference test return

Immediately after completing the RDT, the app asked participants whether they thought they performed the steps of the RDT correctly; 85.9% responded “It was easy to follow and I think I completed the test correctly”, 11.3% noted “It was a little confusing but I think I did the test correctly”, 0.8% noted “It was very confusing and I’m not sure I completed the test correctly”, and 0.6% indicated “During the test, I realized I did something incorrectly” (Additional file 7). Participants were asked the same question about collecting the reference sample; 95.2% said “It was easy to follow and I think I completed the test correctly”, 2% said “It was a little confusing but I think I did the test correctly”, 0.7% indicated “It was very confusing and I’m not sure I completed the test correctly”, and 0.1% said “During the test, I realized I did something incorrectly” (Additional file 8). No adverse events were reported by study participants.

Overall 284 of the 780 returned reference sample packages (36.4%) had errors observed in packaging, including incorrect sealing of the return envelope, biohazard labels missing or applied incorrectly, specimen transport bag not sealed correctly, or damage to the shipping box (Additional file 9). Problems with the reference samples were noted in 180 samples. Most (170) were discolored UTM fluid indicating a spoiled sample, the remaining (10) errors were UTM tube not placed inside transport bag, UTM tube leaking (top loose/missing), nasal swab not in UTM tube, 2 nasal swabs in UTM tube, RDT test strip in UTM tube, or participant entered personal identifying information on the tube label.

### Accuracy of self-test

The sensitivity and specificity of participants’ interpretation of the test strip result compared to the reference test were 14% (95%CI 5–28%) and 90% (95%CI 87–92%), respectively (Table 4). Images of the test strip were uploaded successfully by 96.2% of participants, of these images (*n* = 687), two expert reviewers had consensus on the result for 679 (98.8%) images, and the remaining 8 were reviewed by two additional expert reviewers to make a final determination. The sensitivity and specificity of test strip image interpretation by the research team compared to the reference test were 12% (95%CI 4–25%) and 99% (95%CI 98–100%) respectively. Discrepancy between participant-interpreted results and expert-interpreted results were mainly (57) due to flu-negative participants incorrectly interpreting background red color from the detection particles on the test strip as a positive test. Another source for discrepancy was that 6 image files were unreadable (corrupt file or dark image) for expert review.

**Table 4:** Accuracy of influenza self-test compared to reference specimen PCR

	True positive	False positive	True negative	False negative	Sensitivity (95%CI)	Specificity (95%CI)	PPV (95%CI)	NPV (95%CI)
Participant interpretation of self-test	6	68	603	37	0.14 (0.05, 0.28)	0.90 (0.87, 0.92)	0.08 (0.03, 0.17)	0.94 (0.92, 0.96)
Expert interpretation of image of self-test	5	7	637	38	0.12 (0.04, 0.25)	0.99 (0.98, 1.00)	0.42 (0.15, 0.25)	0.94 (0.92, 0.96)

Test accuracy was similar to the above results among subgroups of individuals who had reported symptom onset of 3 (72 h) or fewer days at the time of testing, those who completed the test within 4 days of ordering, and in those with a reference UTM tube that was not discolored (Additional file 10).

## Discussion

### Main findings

Our study found low sensitivity of a self-test for influenza which involved recruitment of participants with ILI remotely without physical connection to the research team. Sensitivity of this test among the 739 participants self-reporting ILI symptoms (with prevalence of influenza of 5.9%) was only 14%, but specificity in contrast was high (90%). The majority of participants were able to complete the multiple steps required for this study, guided by a mobile app; these included confirming eligibility, consent, guidance in obtaining nasal swabs, and returning a reference sample by mail. Indeed, 93% of individuals completed all steps required for the index test following consent, indicated by reporting RDT test results. However, only about 5% of participants took the test within 48 h of symptom onset, and only an additional 16% took the test within 72 h of symptom onset. Errors due to the user in returning reference test materials by mail were rare (1.3%), and a reference sample was received from the vast majority (95%) of individuals reporting RDT results.

### Comparison to existing literature

Several studies have applied home based sampling using mobile technology to track influenza. For example, a study of influenza surveillance in Japan which used a mobile application to track influenza activity recruited over 10,000 individuals in one influenza season [13]. Web based platforms can have high retention rates over time, even without participant incentives [16]. The Influweb influenza tracking project in Italy assessed ILI in the general population over 4 influenza seasons [17]. Other mobile and web based applications have also provided guidance to participants on how to collect specimens without a trained professional. Two studies, GoViral in the US and Flusurvey in the UK, recruited participants on their web based platforms and successfully demonstrated that participants could self-collect nasal samples [12, 18]. Recent SARS-CoV-2 testing studies also show that self-collected swabs are just as effective as professionally collected swabs [19, 20]. Several point of care tests for SARS-CoV-2 have been evaluated, but mainly in healthcare settings. Conclusions from a systematic review hypothesize that point of care testing in more diverse populations and settings will cause the technical performance of the test to deteriorate [21].

The sensitivity of the influenza RDT used in our study was lower than accuracy reported in two systematic reviews: these reported pooled sensitivity and specificity for the QuickVue Influenza A + B test of 44.6% (95% CI 29.1–60.0%) and 99.3% (95% CI 98.8–99.9%) [22] and 48.8% (95% CI 39.0–58.8%) and 98.4% (95% CI 98.6–99.2%) respectively [23]. In these reviews, there were studies which resembled the results found in our study for QuickVue Influenza A + B (i.e. sensitivity within 15% and specificity within 10%). Their methods included mostly mixed populations of children/adults, sample collection from trained professionals, in some cases recruitment during the 2009 H1N1 pandemic [24–27], and a variety of geographic locations [24–32]. In contrast, our study methods included home study sites, self-sample collection, and an adult-only population during a typical influenza season of our study, making direct comparisons difficult.

We believe that the low observed sensitivity represented time delays from symptom onset to conducting the RDT, although we could not confirm this on subgroup analyses. Viral shedding declines rapidly after 3 days for influenza, yielding samples which are likely below the limit of detection for an antigen-based RDT. The ideal window for testing with RDTs is within 72 h of symptom onset when viral load is at its highest and also in the window when treatment can be administered [33–35]. Low sensitivity may also be partially due to errors in obtaining nasal samples (although this would impact both the index test and the reference test), or conducting the RDT, even though participant self-report suggested such errors were unusual. A final source of diagnostic error was a participant’s ability to correctly read the test strip results. The larger number of false positive results appear to be mainly due to misinterpreted background red color from the detection particles, suggesting interpretation was poor or that participants waited more than 10 min to read their test strip. Specificity increased to 99% when images of the RDT results were interpreted by experts, suggesting that automated systems to interpret RDTs using mobile phone technologies may be valuable [36]. Previous studies have reported that on some lateral flow tests the red test line may appear light and difficult to see, which has been documented to occur for the QuickVue test and may be an indication of lower viral load [34, 37]. Additionally, it is believed that both trained and untrained individuals favor indicating a negative result when the line is faint. This may partially explain the false negative rate.

### Lessons learned

We believe there is much to learn from the study design and procedures that can provide valuable insight for researchers and test developers evaluating the accuracy of self/home tests for influenza and other respiratory viruses such as SARS-CoV-2 (Table 5). This study design allowed us to recruit a large sample of participants nationally through online marketing without face to face clinical sites. However, our sample demographics are limited to majority female and non-Hispanic white, indicating additional recruitment methods are needed for a more diverse sample. Study procedures were guided by an app designed to facilitate multiple steps in the research process, including screening, consent, data collection, and collection of images of the completed test strip to mitigate errors in user interpretation of test strip images. Integrating multiple research procedures and functions within a single digital platform provided a user friendly study interface, and facilitated components such as timers to help guide the participant through steps in the RDT procedure. The single interface also reduced the need to move between on-line study instruments, and took advantage of wait times to administer survey questions. The use of the smartphone to capture an image of the completed test strip also allowed the research team to independently interpret the RDT result. The high rate of completion of the study procedures implies high usability.

**Table 5:** Key lessons learned for design of future evaluations of home tests for influenza and other respiratory pathogens

Study Design Feature	Advantages	Disadvantages
Integrated study functions streamlined into a mobile application	• Reduced resources needed for recruitment • Efficiency in expanding geographic reach • Maintained protocol standardization between participants • Single interface which simplified study procedures and aided participant engagement (e.g. participant took the study questionnaire while they waited for the test results)	• Resources needed to develop app • No human interaction with risks to study fidelity: ➜ Unable to verify accuracy of self-reported responses ➜ Difficult to verify if participants conducted the swabbing correctly without marker of human DNA
Recruitment using online marketing through social media	• Expanded geographic reach • Ability to target specific groups and regions • Improves generalizability (people in the community versus recruitment in a clinic seeking care) • Facilitates tailoring of recruitment materials compared to paper based recruitment materials	• Can be expensive • Financial incentives noted in online recruitment can attract participants only interested in financial reward • Avoid recruitment materials that advertise monetary incentive
Determining eligibility through Self-reported symptoms	• Prevents exposure of study staff to ILI	• Unable to verify eligibility information provided by the participant • We accepted participants with ILI symptoms longer than 72 h
Shipping of test kit to and from study lab	• Prevents exposure of ILI to study staff and potentially people at a health care clinic • Central distribution of study kits allowed for quality control	• Despite priority and overnight shipping, shipping and time to participants taking the test took too long to capture many participants early in their illness
Return of reference sample	• Facilitated reference sampling, without need for study staff or clinic visits • Low error rate and participants reported that it was easy to collect	• Study design did not stress rapid return of the sample thus lead to longer times to return to study lab and spoiled UTM fluid • Time of the year/temperature may impact UTM fluid stability

We identified within the first week of recruitment the study advertisement promoted on websites and social media groups targeted to individuals looking for ways to earn money. Use of financial incentives is more challenging for this type of study than face to face contact with research subjects, and likely led to some individuals participating partly for monetary incentives, and/or who did not meet inclusion criteria. While this can be mitigated to some extent by limiting where the study is advertised online, using minimal financial incentives (or making these less obvious to participants), it is difficult to know how effective these mitigation efforts are. While we believe that a better incentive to participate would have been to display the results of the influenza test to individuals, return of research results from diagnostic tests is prohibited by regulations from state Departments of Health.

Several methods likely impacted test performance. We began recruitment late in the influenza season, thus under-recruited due to decreasing influenza activity, potentially influencing the prevalence of influenza in our sample. Additionally, study recruitment relied on self-report of ILI symptoms which could not be verified. This likely contributed to a lower rate of influenza and milder spectrum of infection, than individuals visiting health care settings, as well as recruitment of participants who did not have ILI. Furthermore, 1 in 10 people did not report cough occurring during their illness on their symptom questionnaire (reported while taking the test) which was an eligibility requirement to be able to order a test kit, indicating some participants may not have been truthful or a long time elapsed between the eligibility and symptom questionnaire. Last, while fever was on the list of eligibility symptoms we did not make it a requirement for participation. Those participating without a fever may have also impacted test sensitivity.

In some instances next day shipping could not be guaranteed (orders after 1 pm and weekends), this contributed to delays in testing. Self-reported time from symptom onset to testing for most participants was 4 or more days, at which time viral load may have been below the limit of detection. Participants’ interpretation of test strips resulted in a large number of false positive test strips. Given that participants were not provided information about the meaning of the test lines, we do not believe this represents participants over-diagnosing influenza, but rather misunderstanding the color changes in test strips. This suggests a need for better guidance for participants, and/or automated ways to interpret test strip images [36]. The reference test relied on individuals collecting and returning a second nasal swab to the reference laboratory. We noted a high rate (21.8%) of spoilage of the UTM fluid based on visual discoloration, which may have adversely affected the reference testing. Uncontrolled temperature conditions can spoil the UTM and/or cause lysis of the virus that exposes viral RNA to enzymes that degrade it. However, removing samples with discolored UTM samples from the analysis did not change test accuracy compared to the overall group. We did not test for a marker of human DNA on the reference swab therefore do not know how well individuals performed the reference or the index swabs.

## Conclusions

This is one of the first studies to measure the accuracy of a test for influenza involving participants conducting both the index and reference tests, unsupervised by the research team. While the test sensitivity we report does not support current deployment of the RDT, several features of the study design have implications for evaluating self tests for influenza and other respiratory viral infections. First, recruiting study participants within windows of infectivity where not only is viral material within the expected limit of detection of the RDT, but also when a test result could lead to an actionable result is critical. This can be done through restricting time from symptom onset in the inclusion criteria. The multiple strengths of using a mobile app platform (streamlined study procedures etc.) need to be balanced by its inherent weaknesses (lack of face to face contact with research team). Optimize user interpretation of test results using automated interpretation where possible. Minimize delays in delivering test kits by pre-positioning tests at local clinics prior to a study or at healthy participants’ homes prior to an illness, or reducing a study’s geographic reach. Last, improve the speed of return and/or improve the stability of reference samples.

## Supplementary Information

**Additional file 1.** Description of Audere, app design and operation.

**Additional file 2.** Study Questionnaire.

**Additional file 3.** Recruitment sources of study participants. Table of recruitment sources - N (%): Overall, PCR +, PCR –.

**Additional file 4.** Count of symptoms among participants with and without influenza. Table of number of symptom - N (%): Overall, PCR +, PCR –.

**Additional file 5.** Self-reported symptom severity. Table of symptom severity broken out by symptom - N (%): Overall, PCR +, PCR –.

**Additional file 6.** Onset of symptoms in participants with and without influenza. Table of onset of symptom - N (%): Overall, PCR +, PCR –.

**Additional file 7.** Participant response to “Do you feel you performed all of the steps in the flu test correctly”. Table of response options - N (%): Overall, PCR +, PCR –.

**Additional file 8.** Participant response to “How do you feel you performed the second test?”. Table of response options - N (%): Overall, PCR +, PCR –.

**Additional file 9.** Documented kit errors upon receipt at study laboratory. 3 Tables: Number of missing barcodes from returned kits; Errors reported on sample and shipping packaging; Reference sample reported errors.

**Additional file 10.** Secondary diagnostic accuracy analysis. 3 Tables: Accuracy of user interpreted self-test and Expert interpreted self-test results removing samples with discolored UTM fluid; Accuracy of user interpreted self-test and Expert interpreted self-test results with a subset of participants that took the test between 0 and 4 days of ordering it; Accuracy of user interpreted self-test and Expert interpreted self-test results with a subset of participants who symptom onset ≤3 days.

## Data Availability

The datasets generated during the current study are not publicly available due to additional planned analyses for the dataset and IRB restrictions but are available from the corresponding author on reasonable request and proof of IRB approval from requesting party.

## Abbreviations

US: United States
ILI: Influenza-like-illness
RDT: Rapid diagnostic test
UTM: Universal transport medium
PCR: Polymerase chain reaction
Ct: Cycle threshold
CI: Confidence interval
