# Supplementary material for: Evaluating an app-guided self-test for influenza: lessons learned for improving the feasibility of study designs to evaluate self-tests for respiratory viruses
Source: BMC Infect Dis. 2021 Jun 29;21:617. doi: 10.1186/s12879-021-06314-1 (PMC8240430; doi:10.1186/s12879-021-06314-1)
Supplement: Supplementary file 1 — Additional file 1. Description of Audere, app design and operation. [file 12879_2021_6314_MOESM1_ESM.docx]

# **Additional file 1: Description of Audere, app design and operation**

Audere is a Seattle-based digital health nonprofit developing software to improve global health in the world’s most underserved communities. Our team of passionate, innovative minds combines smartphone technology, computer vision & machine learning, and the best of cloud-based services to deliver healthcare technology solutions worldwide. Development of our projects is funded by grants from the Bill & Melinda Gates Foundation and Justworks. Learn more at auderenow.org.

The flu@home app was available on iPhones and iPads. The app was available in English for the study. However, the app supports development and release of other languages as needed. The entire app experience used a touch-sensitive dynamic interface on the device. The app ensured the proper test procedure was followed through clear instructions and timers that prevented the participant from moving forward in the test process until the time was right (e.g., ensuring the test strip remained in the test fluid for a full ten minutes to be certain the strip had enough time to process before reading the result). The app attempted to keep participants engaged during wait times by providing flu-related informational facts (during an initial one-minute timer when the nasal swab was processing in the RDT vial) and asking the participant to answer a set of demographic and illness-related survey questions (during the ten-minute timer). Field-level validation was employed to ensure participants answered specific required questions in the survey. The app was built using React Native, a JavaScript framework used to create mobile applications for iOS. The app communicated with the Google Cloud Platform (Firebase) to queue survey data and Firebase Storage to queue images captured of the RDT. These were pulled by a NodeJS service into a PostgreSQL database hosted on an AWS Relational Database Service (RDS), which allowed for operation and scale of a relational database in the cloud. App data was stored in Amazon Web Services (AWS S3 and AWS RDS). Amazon Simple Store Service (S3) provided a straightforward web services interface that was used to store and retrieve data, such as PCR data used for comparison to the RDT test results. Access to S3 requires user authentication. From the time data left the client, all data was encrypted both at rest and over communication links. Audere used AWS Key Management Service (KMS) to encrypt data at rest in AWS, and Google Cloud Platform automatically encrypted its data using Advanced Encryption Standard (AES). All connections to the app occurred over Secure Sockets Layer (SSL), a standard security technology that established an encrypted link between a web server and browser, ensuring all data traversing the web server and browser remained private. For near real-time reporting, Metabase was run in an Elastic Container Service (ECS) in the same AWS project referencing the same app data.

The app used Firebase caching and analytics to track each participant page view, including a timestamp for each page view. Firebase was also used to track changed answers if a participant navigated back in the app flow.
