# Supplementary material for: Evaluating an app-guided self-test for influenza: lessons learned for improving the feasibility of study designs to evaluate self-tests for respiratory viruses
Source: BMC Infect Dis. 2021 Jun 29;21:617. doi: 10.1186/s12879-021-06314-1 (PMC8240430; doi:10.1186/s12879-021-06314-1)
Supplement: Supplementary file 2 — Additional file 2. Study Questionnaire. [file 12879_2021_6314_MOESM2_ESM.docx]

# **Additional file 2: Eligibility Questionnaire and Study Questionnaire**

Eligibility Questionnaire

What do I need to do?

We need to ask a few questions about you and the symptoms you’re feeling. In addition, you will need to provide your home or mailing address so we can send the flu kit to you.

Question 1:

How old are you? (select one)

- 17 and under
- 18 to 24
- 25 to 34
- 35 to 44
- 45 to 64
- 65 and older

Question 2:

Describe your symptoms (please select all that apply)

- Feeling feverish
- Feeling chills or sweats
- Coughing up anything
- Feeling tired or more tired than usual
- Muscle or body aches
- None of the above

Study Questionnaire Conducted in App While Taking the Self-test

***Symptom Survey**

*Questions marked with an * are required.*

| **Symptom** | ***Which of the following were present during your illness?** | ***How long ago did symptoms start? (Select the time frame that best applies)** | ***Were these symptoms present in the last 48 hours?** | ***How severe were your symptoms? (Select the level of discomfort you felt at the worst point)** |
| --- | --- | --- | --- | --- |
| Fever | YES/NO | 1 day, 2 days, 3 days, 4+ days | YES/NO | mild, moderate, severe |
| Chills or sweats | YES/NO | 1 day, 2 days, 3 days, 4+ days | YES/NO | mild, moderate, severe |
| Cough | YES/NO | 1 day, 2 days, 3 days, 4+ days | YES/NO | mild, moderate, severe |
| Sore throat | YES/NO | 1 day, 2 days, 3 days, 4+ days | YES/NO | mild, moderate, severe |
| Headache | YES/NO | 1 day, 2 days, 3 days, 4+ days | YES/NO | mild, moderate, severe |
| Feeling tired or more tired than usual | YES/NO | 1 day, 2 days, 3 days, 4+ days | YES/NO | mild, moderate, severe |
| Muscle or body aches | YES/NO | 1 day, 2 days, 3 days, 4+ days | YES/NO | mild, moderate, severe |
| Runny or stuffy nose | YES/NO | 1 day, 2 days, 3 days, 4+ days | YES/NO | mild, moderate, severe |
| Shortness of breath | YES/NO | 1 day, 2 days, 3 days, 4+ days | YES/NO | mild, moderate, severe |

**General exposure**

In the next section, the questions are going to be about being **in contact** with people who seemed to have a cold. **In contact** means being within six feet of them for at least two minutes or physical contact for any amount of time.

- *For reference, six feet is about the distance between you and someone sitting two rows ahead of you on the bus.*

**In the past week, have you been in contact with a person who seemed to have a cold?**

- Yes
- No
- Don’t know

**[If YES] Were they coughing or sneezing?**

- Yes
- No
- Don’t know

**In the past week, have you been in contact with any children under five years old for over an hour?**

- No contact with children under 5 yrs
- 1 child
- 2-5 children
- More than 5 children
- Don’t know

**Are there any children under 18 years old in your household?**

- Yes
- No

**[If YES] Do any children in your household attend a school, childcare setting, or play group with at least three other children for a total of three or more hours per week?**

- Yes
- No
- Don’t know

**How many people live in your household (including you)?**

- 1-2
- 3-4
- 5-7
- 8+

**How many bedrooms are in your home?**

- 0-1
- 2
- 3
- 4
- 5+

***General Health***

*Next we’d like to ask you some questions about your overall health:*

**Have you ever been told by a doctor that you have one of the following medical conditions? (SELECT ALL THAT APPLY)**

- Asthma
- COPD/emphysema
- Diabetes
- None of these
- Do not know

**Did you get a flu shot in the last year?**

- Yes
- No
- Do not know

**[If YES] what was the date of your flu shot?**

- Choose month and year
  [Programming note: We need Jan 2018 - current month for the choose month/year options]

**Do you smoke tobacco?**

- Yes
- No

**Does anyone in your household smoke tobacco?**

- Yes
- No

**Is your illness preventing you from going to work or school, going to social events, or exercising/working out?**

- Yes
- No

***Are you currently taking antibiotics or antivirals prescribed by a doctor for this illness?**

- Yes
- No
- Do not know

**What is the sex on your medical records?**

- Male
- Female
- Other

**How would you describe your race?** Please select all that apply.

- American Indian or Alaska Native
- Asian
- Native Hawaiian or Other Pacific Islander
- Black or African American
- White
- Other
- Prefer not to say

**Are you Hispanic or Latino?**

- Yes
- No
- Prefer not to say

**What kind of medical insurance do you have? .** Please select all that apply
[Programming note: Multi-select, except if “I do not have medical insurance” is selected that’s the only one that can be selected.]

- I do not have medical insurance
- Insurance from my employer
- Insurance I purchased myself
- Medicaid or Medicare
- Do not know
- Prefer not to say
- Other

[Then the app takes the participant back to the RDT test which has now completed its 10 min wait period.]

**Nice job! Do you feel you performed all of the steps in the flu test correctly? [Note this is after Quidel test] Check all that apply**

- It was easy to follow and I think I completed the test correctly
- It was a little confusing but I think I did the test correctly
- It was very confusing and I’m not sure I completed the test correctly
- During the test, I realized I did something incorrectly

[After UTM test]

**Thanks, you did it! How do you feel you performed the second test?**

- It was easy to follow and I think I completed the test correctly
- It was a little confusing but I think I did the test correctly
- It was very confusing and I’m not sure I completed the test correctly
- During the test, I realized I did something incorrectly

**Follow-up Questionnaire (1 week later)**

Thank you for taking part in the Flu@Home Study!

We want to ask you just 3 quick questions about your illness over the last 7 days. This will take no more than 2 minutes to complete.

**Over the last week, did your illness prevent you from going to work or school, going to a social event, or exercising/working out?**

- Yes
- No

**Are you currently taking antibiotics or antivirals prescribed by a doctor for this illness?**

- Yes
- No
- Do not know

**In the last week did you go to any of the following for health care treatment or advice about your illness? (select any that you attended, or received care or advice from)**

- Pharmacy
- Primary care clinic
- Urgent care clinic
- Naturopath
- Online health care provider
- Emergency department
- Other
- None of the above
