# Supplementary material for: Evaluating an app-guided self-test for influenza: lessons learned for improving the feasibility of study designs to evaluate self-tests for respiratory viruses
Source: BMC Infect Dis. 2021 Jun 29;21:617. doi: 10.1186/s12879-021-06314-1 (PMC8240430; doi:10.1186/s12879-021-06314-1)
Supplement: Supplementary file 3 — Additional file 3. Recruitment sources of study participants. Table of recruitment sources - N (%): Overall, PCR +, PCR –. [file 12879_2021_6314_MOESM3_ESM.docx]

# **Additional file 3: Recruitment sources of study participants**

| **Sources** | **N (%)**  **(N = 739)** | **PCR+**  **(N=43)** | **PCR-**  **(N=696)** |
| --- | --- | --- | --- |
| No response | 187 (25.3) | 9 (20.9) | 178 (25.6) |
| App Store | 6 (0.8) | 0 | 6 (0.9) |
| Coupon Site | 6 (0.8) | 0 | 6 (0.9) |
| Flu Near You | 46 (6.2) | 3 (6.9) | 43 (6.2) |
| Friend | 91 (12.3) | 6 (13.9) | 85 (12.2) |
| Local Clinic | 6 (0.8) | 2 (4.7) | 4 (0.6) |
| Online Advertisement | 218 (29.5) | 16 (37.2) | 202 (29.0) |
| Online Search | 118 (15.9) | 4 (9.3) | 114 (16.4) |
| Other | 61 (8.3) | 3 (6.9) | 58 (8.3) |
