# Supplementary material for: Evaluating an app-guided self-test for influenza: lessons learned for improving the feasibility of study designs to evaluate self-tests for respiratory viruses
Source: BMC Infect Dis. 2021 Jun 29;21:617. doi: 10.1186/s12879-021-06314-1 (PMC8240430; doi:10.1186/s12879-021-06314-1)
Supplement: Supplementary file 4 — Additional file 4. Count of symptoms among participants with and without influenza. Table of number of symptom - N (%): Overall, PCR +, PCR –. [file 12879_2021_6314_MOESM4_ESM.docx]

# **Additional file 4: Count of symptoms among participants with and without influenza**

| **# of Symptoms** | **N (%)**  **(N=739)** | **PCR +**  **(N = 43)** | **PCR –**  **(N = 696)** | **p-value** |
| --- | --- | --- | --- | --- |
| 1 | 2 (0.3) | 0 (0) | 2 (0.3) | 0.041 |
| 2 | 8 (1.1) | 0 (0) | 8 (1.1) |  |
| 3 | 19 (2.6) | 0 (0) | 19 (2.8) |  |
| 4 | 43 (5.8) | 1 (2.3) | 42 (6.0) |  |
| 5 | 84 (11.4) | 4 (9.3) | 70 (11.5) |  |
| 6 | 123 (16.6) | 2 (4.7) | 121 (17.5) |  |
| 7 | 158 (21.4) | 6 (13.9) | 152 (21.8) |  |
| 8 | 158 (21.4) | 15 (34.9) | 143 (20.6) |  |
| 9 | 144 (19.5) | 15 (34.9) | 129 (18.5) |  |
