# Supplementary material for: Evaluating an app-guided self-test for influenza: lessons learned for improving the feasibility of study designs to evaluate self-tests for respiratory viruses
Source: BMC Infect Dis. 2021 Jun 29;21:617. doi: 10.1186/s12879-021-06314-1 (PMC8240430; doi:10.1186/s12879-021-06314-1)
Supplement: Supplementary file 5 — Additional file 5. Self-reported symptom severity. Table of symptom severity broken out by symptom - N (%): Overall, PCR +, PCR –. [file 12879_2021_6314_MOESM5_ESM.docx]

# **Additional file 5: Self-reported symptom severity**

| **Symptoms and Severity** | **N (%)** | **PCR +** | **PCR -** | **p-values** |
| --- | --- | --- | --- | --- |
| **Fever (N:440)** | | | | |
| Severity: mild | 121 (27.5) | 9 (24.3) | 112 (28.8) | 0.7951 |
| Severity: moderate and severe | 319 (72.5) | 28 (75.7) | 228 (72.2) |  |
| **Cough (N:671)** | | | | |
| Severity: mild | 131 (19.5) | 4 (9.5) | 127 (20.2) | 0.136 |
| Severity: moderate and severe | 540 (80.5) | 38 (90.5) | 502 (79.8) |  |
| **Fatigue (N:686)** | | | | |
| Severity: mild | 57 (8.4) | 1 (2.4) | 56 (8.7) | 0.250 |
| Severity: moderate and severe | 629 (91.6) | 41 (97.6) | 588 (91.3) |  |
| **Chills or swats (N:518)** | | | | |
| Severity: mild | 109 (21.0) | 4 (9.8) | 105 (22.0) | 0.099 |
| Severity: moderate and severe | 409 (79.0) | 37 (90.2) | 372 (78.0) |  |
| **Muscle or body ache (N:581)** | | | | |
| Severity: mild | 69 (11.9) | 2 (5.4) | 67 (12.4) | 0.319 |
| Severity: moderate and severe | 512 (88.1) | 20 (54.1) | 284 (52.2) |  |
| **Sore throat (N:570)** | | | | |
| Severity: mild | 124 (21.8) | 11 (31.4) | 113 (21.1) | 0.222 |
| Severity: moderate and severe | 446 (78.2) | 24 (68.6) | 422 (78.9) |  |
| **Headache (N:615)** | | | | |
| Severity: mild | 89 (14.5) | 3 (8.6) | 86 (14.8) | 0.438 |
| Severity: moderate and severe | 526 (85.5) | 32 (91.4) | 494 (85.2) |  |
| **Runny nose (N:657)** | | | | |
| Severity: mild | 75 (11.4) | 6 (15.4) | 69 (11.2) | 0.586 |
| Severity: moderate and severe | 582 (88.6) | 33 (84.6) | 549 (88.8) |  |
| **Shortness of breath (N:333)** | | | | |
| Severity: mild | 87 (26.1) | 6 (24.0) | 81 (26.3) | 0.988 |
| Severity: moderate and severe | 246 (73.9) | 19 (76.0) | 227 (73.7) |  |
