# Supplementary material for: Evaluating an app-guided self-test for influenza: lessons learned for improving the feasibility of study designs to evaluate self-tests for respiratory viruses
Source: BMC Infect Dis. 2021 Jun 29;21:617. doi: 10.1186/s12879-021-06314-1 (PMC8240430; doi:10.1186/s12879-021-06314-1)
Supplement: Supplementary file 6 — Additional file 6. Onset of symptoms in participants with and without influenza. Table of onset of symptom - N (%): Overall, PCR +, PCR –. [file 12879_2021_6314_MOESM6_ESM.docx]

# **Additional file 6: Onset of symptoms in participants with and without influenza**

| **Days** | **N (%)**  **N=739** | **PCR +**  **N=43** | **PCR –**  **N=696** | **p-value** |
| --- | --- | --- | --- | --- |
| 1-2 | 36 (4.9) | 1 (2.3) | 35 (5.0) | 0.024 |
| 3 | 116 (15.7) | 13 (30.2) | 101 (14.8) |  |
| **≥**4 | 587 (79.4) | 29 (67.4) | 687 (80.2) |  |
