# Supplementary material for: Evaluating an app-guided self-test for influenza: lessons learned for improving the feasibility of study designs to evaluate self-tests for respiratory viruses
Source: BMC Infect Dis. 2021 Jun 29;21:617. doi: 10.1186/s12879-021-06314-1 (PMC8240430; doi:10.1186/s12879-021-06314-1)
Supplement: Supplementary file 7 — Additional file 7. Participant response to “Do you feel you performed all of the steps in the flu test correctly”. Table of response options - N (%): Overall, PCR +, PCR –. [file 12879_2021_6314_MOESM7_ESM.docx]

# **Additional file 7: Participant response to “Do you feel you performed all of the steps in the flu test correctly”**

| **QuickVue Test** | **N (%)** | **PCR +** | **PCR -** | **p-value** |
| --- | --- | --- | --- | --- |
| It was a little confusing but I think I did the test correctly | 84 (11.4) | 3 (6.9) | 81 (11.6) | 0.553 |
| It was very confusing and I’m not sure I completed the test correctly | 7 (0.9) | 0 (0) | 7 (1.0) |  |
| It was easy to follow and I think I completed the test correctly | 637 (86.2) | 40 (93.0) | 594 (85.3) |  |
| During the test, I realized I did something incorrectly | 4 (0.5) | 0 (0) | 4 (0.6) |  |
| No Response | 7 (0.9) | 0 (0) | 10 (1.4) |  |
