# Supplementary material for: Evaluating an app-guided self-test for influenza: lessons learned for improving the feasibility of study designs to evaluate self-tests for respiratory viruses
Source: BMC Infect Dis. 2021 Jun 29;21:617. doi: 10.1186/s12879-021-06314-1 (PMC8240430; doi:10.1186/s12879-021-06314-1)
Supplement: Supplementary file 8 — Additional file 8. Participant response to “How do you feel you performed the second test?”. Table of response options - N (%): Overall, PCR +, PCR –. [file 12879_2021_6314_MOESM8_ESM.docx]

# **Additional file 8: Participant response to “How do you feel you performed the second test?”**

| **Reference Sample** | **N (%)** | **PCR +** | **PCR -** | **p-value** |
| --- | --- | --- | --- | --- |
| It was a little confusing but I think I did the test correctly | 15 (2.0) | 1 (2.3) | 14 (2.0) | 0.562 |
| It was very confusing and I’m not sure I completed the test correctly | 5 (0.7) | 0 (0) | 5 (0.7) |  |
| It was easy to follow and I think I completed the test correctly | 704 (95.2) | 42 (97.7) | 662 (95.1) |  |
| During the test, I realized I did something incorrectly | 1 (0.1) | 0 (0) | 1 (0.1) |  |
| No Response | 14 (1.9) | 0 (0) | 14 (2.0) |  |
