# Supplementary material for: Evaluating an app-guided self-test for influenza: lessons learned for improving the feasibility of study designs to evaluate self-tests for respiratory viruses
Source: BMC Infect Dis. 2021 Jun 29;21:617. doi: 10.1186/s12879-021-06314-1 (PMC8240430; doi:10.1186/s12879-021-06314-1)
Supplement: Supplementary file 9 — Additional file 9. Documented kit errors upon receipt at study laboratory. 3 Tables: Number of missing barcodes from returned kits; Errors reported on sample and shipping packaging; Reference sample reported errors. [file 12879_2021_6314_MOESM9_ESM.docx]

# **Additional file 9: Documented kit errors upon receipt at study laboratory**

**Number of missing barcodes from returned kits**

| **Barcode location** | **N (%) Missing** |
| --- | --- |
| Box | 19 (2.4%) |
| RDT | 4 (0.5%) |
| UTM | 1 (0.1%) |
| Test Strip | 14 (1.8%) |

**Errors reported on sample and shipping packaging (N=284 shipping errors)**

| **Error Type** | **N(%)** |
| --- | --- |
| Shipper box missing (shipped in bag or other) | 4 (1.4%) |
| Shipper box damaged | 12 (4.2%) |
| Shipper box not closed properly | 3 (1.1%) |
| Tape strip backing not removed | 61 (21.5%) |
| UN3373 sticker missing or applied incorrectly | 27 (9.5%) |
| Address label applied incorrectly | 3 (1.1%) |
| Shipper box barcode (blue) missing | 8 (2.8%) |
| Specimen transport bag not sealed correctly | 223 (78.5%) |
| Specimen transport bag missing | 2 (0.7%) |

**Reference sample reported errors (N=180 UTM errors)**

| **Error Type** | **N(%)** |
| --- | --- |
| UTM tube not inside large transport bag | 3 (1.7%) |
| UTM tube leaked, top loose/missing | 1 (0.6%) |
| Swab not in UTM tube | 4 (2.2%) |
| 2 swabs in UTM tube | 3 (1.7%) |
| RDT test strip in UTM tube | 1 (0.6%) |
| UTM fluid discolored | 170 (94.4%) |
| User filled out the blank label | 1 (0.6%) |
