# Supplementary material for: Evaluating an app-guided self-test for influenza: lessons learned for improving the feasibility of study designs to evaluate self-tests for respiratory viruses
Source: BMC Infect Dis. 2021 Jun 29;21:617. doi: 10.1186/s12879-021-06314-1 (PMC8240430; doi:10.1186/s12879-021-06314-1)
Supplement: Supplementary file 10 — Additional file 10. Secondary diagnostic accuracy analysis. 3 Tables: Accuracy of user interpreted self-test and Expert interpreted self-test results removing samples with discolored UTM fluid; Accuracy of user interpreted self-test and Expert interpreted self-test results with a subset of participants that took the test between 0 and 4 days of ordering it; Accuracy of user interpreted self-test and Expert interpreted self-test results with a subset of participants who symptom onset ≤3 days. [file 12879_2021_6314_MOESM10_ESM.docx]

# **Additional file 10: Secondary diagnostic accuracy analysis**

**Accuracy of user interpreted self-test and Expert interpreted self-test results removing samples with discolored UTM fluid**

|  | **True positive** | **False positive** | **True negative** | **False negative** | **Sensitivity (95%CI)** | **Specificity (95%CI)** | **PPV (95%CI)** | **NPV (95%CI)** |
| --- | --- | --- | --- | --- | --- | --- | --- | --- |
| Participant interpretation of self-test | 5 | 50 | 484 | 34 | 0.13 (0.04, 0.27) | 0.91 (0.88, 0.93) | 0.09 (0.03, 0.20) | 0.93 (0.91, 0.95) |
| Expert interpretation of image of self-test | 5 | 6 | 506 | 34 | 0.13 (0.04, 0.27) | 0.99 (0.97, 1.00) | 0.45 (0.17, 0.77) | 0.94 (0.91, 0.96) |

**Accuracy of user interpreted self-test and Expert interpreted self-test results with a subset of participants that took the test between 0 – 4 days of ordering it**

|  | **True positive** | **False positive** | **True negative** | **False negative** | **Sensitivity (95%CI)** | **Specificity (95%CI)** | **PPV (95%CI)** | **NPV (95%CI)** |
| --- | --- | --- | --- | --- | --- | --- | --- | --- |
| Participant interpretation of self-test | 5 | 53 | 505 | 35 | 0.12 (0.04, 0.27) | 0.91 (0.88, 0.93) | 0.09 (0.03, 0.19) | 0.94 (0.91, 0.95) |
| Expert interpretation of image of self-test | 4 | 6 | 535 | 36 | 0.10 (0.03, 0.24) | 0.99 (0.98, 1.00) | 0.40 (0.12, 0.74) | 0.94 (0.91, 0.96) |

**Accuracy of user interpreted self-test and Expert interpreted self-test results with a subset of participants who symptom onset ≤ 3 days**

|  | **True positive** | **False positive** | **True negative** | **False negative** | **Sensitivity (95%CI)** | **Specificity (95%CI)** | **PPV (95%CI)** | **NPV (95%CI)** |
| --- | --- | --- | --- | --- | --- | --- | --- | --- |
| Participant interpretation of self-test | 2 | 12 | 120 | 12 | 0.14 (0.02, 0.43) | 0.91 (0.85, 0.95) | 0.14 (0.02, 0.43) | 0.91 (0.85, 0.95) |
| Expert interpretation of image of self-test | 1 | 3 | 129 | 13 | 0.07 (0.00, 0.34) | 0.98 (0.94, 1.00) | 0.25 (0.01, 0.81) | 0.91 (0.85, 0.95) |
